# News and Coming Events

**Published:** 1907-05-04

**Authors:** 


### NEWS AND COMING EVENTS.

A new hospital for infectious diseases has been opened at Peterhead.

Lord Armstrong and Sir Riley Lord are appealing for funds on behalf of the Royal Victoria Infirmary, Newcastle-on-Tyne.

Lord Northcliffe presides at the 86th anniversary festival of the Printers' Pension, Almshouse, and Orphan Asylum Corporation, to be held at the Hotel Cecil on May 29.

The annual meeting of Governors of the Chelsea Hospital for Women will be held on Tuesday, May 14, at 4 p.m. The President (the Right Hon. the Lord Glenesk) will be in the chair.

Mr. F. Brinsley-Harper, C.C., has been elected a member of the Committee of Management of the City of London Hospital for Diseases of the Chest (Victoria Park Hospital), Victoria Park, E.

H.R.H. the Prince of Wales will preside at the dinner of the National Association for the establishment of sanatoria for workers suffering from tuberculosis, to be held at the Hotel Cecil on Tuesday, May 14.

The annual meeting of the City of London Hospital for Diseases of the Chest will be held on Thursday, May 9, at 3.30 p.m., at Gresham College, Basinghall Street, E.C. Sir E. Sassoon, Bart., M.P., Treasurer of the Hospital, will preside.

Mr. R. Rainy, M.P., will deliver an address on "The Necessity for a Minister of Public Health" at the rooms of the New Reform Club, 10 Adelphi Terrace, W.C., on Friday, May 10, at 8.30 p.m. Sir William Collins will be in the chair.

The Jacksonian Prize of the Royal College of Surgeons for the year 1906, on "The Diagnosis and Treatment of those Diseases and Morbid Growths of the Vertebral Column, Spinal Cord, and Canal which are amenable to Surgical Operations," has been awarded to Mr. Donald John Armour, F.R.C.S.

Mr. H. W. Burleigh, Assistant Secretary of the Royal Sea-bathing Hospital, has been appointed to succeed Mr. H. Howgrave Graham as Secretary to the Hospital for Nervous Diseases, Maida Vale. Mr. H. W. Burleigh has been actively connected with the Hospital Officers' Association since its foundation, has served on its Council, and has acted as honorary editor of the *Hospital Gazette*. Mr. Burleigh has shown exemplary zeal in making himself familiar with the needs and work of London hospitals, and we wish him every success.

A new building for the Victoria Dental Hospital, Manchester, is to be erected on the west side of Oxford Road, near the University. For more than 20 years, owing largely to lack of funds, the hospital has been housed in temporary premises, first in Grosvenor Street and until lately in Devonshire Street, All Saints. These premises are required by the Corporation for education purposes, and it has been decided to put up a permanent hospital, with full and up-to-date arrangements both for the treatment of patients and the training of students. The cost of the building, with its equipment, will be between £12,000 and £15,000.

Major Leonard Rogers, I.M.S., will deliver the inaugural lecture of the summer session at the Post-Graduate College, West London Hospital, on Monday, May 13, at 5 p.m. The lecture will be on the "Clinical Diagnosis of Fevers in the Tropics," and will be illustrated by lantern slides.

On Princess Mary's tenth birthday, Thursday, April 25, Her Royal Highness the Princess of Wales sent a gift of primroses to the patients of the Royal Hospital for Incurables, Putney Heath. Her Royal Highness has for some years been much interested in the work of that institution. Each of the 214 patients received some of the flowers, which were distributed by the matron.

The Annual Court of Governors of the Evelina Hospital for Children was held on Wednesday (April 24). Mr. Charles Wightman presided, and submitted the 37th annual report. The chief feature of the year has been the construction of the new out-patient department, which is now completed; the committee were forced to carry out this improvement so as to cope efficiently with the growing demands on the institution. There is yet a debt of about £7,000 on the new buildings. The Lord Mayor will open the new out-patient department of the hospital on May 23.

Lord Kinnaird presided at the annual meeting of the Zenana Bible and Medical Mission on Friday, April 26. Mr. Richard Sorabji, a native of India, paid a high tribute to the work carried on by the missionaries. The Society now had 431 European and Indian workers in the field; 51 schools and institutions in which there are 2,595 pupils; as well as 328 women and girls in the orphanages. At the hospitals and dispensaries in Lucknow, Benares, Patna, Nasik, Ajoudhya, and Jaunpur there were 2,066 in-patients, 29,595 out-patients, 231 were attended at home, and the attendances at dispensaries numbered 83,494.

Lord Stanley makes an appeal on behalf of the Liverpool Samaritan Hospital for Women. The number of beds in this hospital is quite inadequate, and patients are frequently obliged to wait several weeks before they are admitted. A scheme to cost about £350 providing for necessary improvements is at the present time under consideration by the committee. The committee makes a special appeal to the public for a sum of £1,600, which would enable it to deal with the need for additional accommodation, to liquidate the debt, and to pay off the mortgage. Mr. W. P. Hartley has promised £400 to this special fund, on condition that the balance of the amount is raised within the next few months.

Much regret has been caused by the death of Mr. P. B. Wooldridge, who has been connected with Charing Cross Hospital for several years in the secretarial department. His body was washed ashore at Ramsgate, and it appears from evidence given at the inquest that Mr. Wooldridge was in financial difficulties, though the amount was relatively small. The jury returned a verdict of "Temporary Insanity." It seems to us that the Hospital Officers' Association should provide the means to secure that in future no member need suffer unnecessary anxiety through temporary financial trouble, because he should possess the privilege of explaining his troubles to the President with a view to their adjustment.

